# Custom-made holey graphene *via* scanning probe block co-polymer lithography†

**DOI:** 10.1039/d1na00769f

**Published:** 2022-01-31

**Authors:** Samar A. Alsudir, Roa S. Fardous, Shahla Alsoughayer, Abdulaziz M. Almalik, Edreese H. Alsharaeh, Ali H. Alhasan

**Affiliations:** National Center for Pharmaceutical Technology, Life Science and Environmental Research Institute, King Abdulaziz City for Science and Technology (KACST) P. O. Box 6086 Riyadh 11461 Saudi Arabia; KACST-BWH/Harvard Centre of Excellence for Biomedicine, Joint Centers of Excellence Program, King Abdulaziz City for Science and Technology (KACST) P. O. Box 6086 Riyadh 11461 Saudi Arabia; College of Science and General Studies, Alfaisal University P. O. Box 50927 Riyadh 11533 Saudi Arabia; National Center for Biotechnology, Life Science and Environmental Research Institute, King Abdulaziz City for Science and Technology (KACST) P. O. Box 6086 Riyadh 11461 Saudi Arabia aalhasan@kacst.edu.sa

## Abstract

Oxidative chemical etching of metal nanoparticles (NPs) to produce holey graphene (hG) suffers from the presence of aggregated NPs on the graphene surface triggering heterogeneous etching rates and thereby producing irregular sized holes. To encounter such a challenge, we investigated the use of scanning probe block co-polymer lithography (SPBCL) to fabricate precisely positioned silver nanoparticles (AgNPs) on graphene surfaces with exquisite control over the NP size to prevent their aggregation and consequently produce uniformly distributed holes after oxidative chemical etching. SPBCL experiments were carried out *via* printing an ink suspension consisting of poly(ethylene oxide-*b*-2-vinylpyridine) and silver nitrate on a graphene surface in a selected pattern under controlled environmental and instrumental parameters followed by thermal annealing in a gaseous environment to fabricate AgNPs. Scanning electron microscopy revealed the uniform size distribution of AgNPs on the graphene surface with minimal to no aggregation. Four main sizes of AgNPs were obtained (37 ± 3, 45 ± 3, 54 ± 2, and 64 ± 3 nm) *via* controlling the printing force, *z*-piezo extension, and dwell time. Energy dispersive X-ray spectroscopy analysis validated the existence of elemental Ag on the graphene surface. Subsequent chemical etching of AgNPs using nitric acid (HNO_3_) with the aid of sonication and mechanical agitation produced holes of uniform size distribution generating hG. The obtained *I*_D_/*I*_G_ ratios ≤ 0.96 measured by Raman spectroscopy were lower than those commonly reported for GO (*I*_D_/*I*_G_ > 1), indicating the removal of more defective C atoms during the etching process to produce hG while preserving the remaining C atoms in ordered or crystalline structures. Indeed, SPBCL could be utilized to fabricate uniformly distributed AgNPs of controlled sizes on graphene surfaces to ultimately produce hG of uniform hole size distribution.

## Abstract 2

Scanning probe block co-polymer lithography was utilized to fabricate silver nanoparticles on graphene surfaces with spatial and dimensional control. The downstream etching process produced holey graphene with uniformly distributed holes.

## Graphical abstract

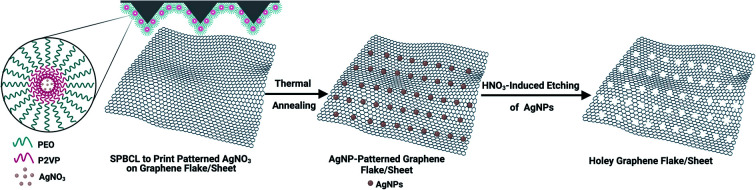

## Introduction

Among various carbon nanostructures, holey graphene (hG) has attracted considerable interest owing to its exceptional properties including excellent electrical conductivity, large specific surface area, and superior chemical/electrochemical stability surpassing its pristine graphene form.^1,2^ It is synthesized *via* perforating abundant in-plane pores using either bottom-up^3–5^ or top-down^6–8^ methodologies, among which the so-called template-assisted or template-free approaches are the most common. The latter approach operates *via* oxidative chemical etching of graphene using either oxidants such as H_2_O_2_,^6,7^ O_2_,^9^ nitric acid (HNO_3_),^10^ and potassium hydroxide (KOH),^11^ or catalysts including metal (Ag^12–14^ and Cu^15^) or metal oxide (ZnO,^16^ MnO_2_,^17^ SnO_2_,^18^ and Fe_2_O_3_)^19^ nanoparticles (NPs). The catalytic etching process involves NP deposition on the graphene basal plane, etching reaction, and catalyst removal. The deposited NPs could catalytically oxidize C atoms to produce nano-sized holes under specific conditions such as annealing or photochemical/sonochemical treatments. Although graphene is used as a mesh to enhance NP distribution, aggregation still persists,^12,20–23^ which is a hammering obstacle resulting in heterogeneous etching rates^24,25^ and thereby producing irregular sized holes.^15,26^ Therefore, advanced methodologies for fabricating hG with tunable hole sizes are coveted.

**Scheme 1:**
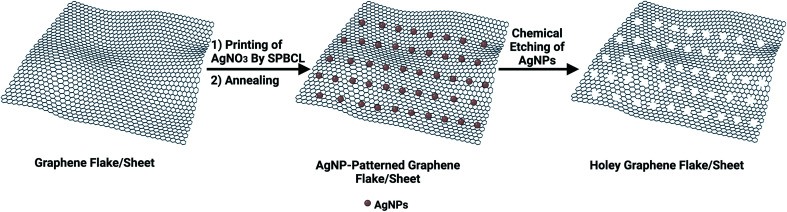
Proposed SPBCL route to fabricate hG.

Inspired by the successful route of scanning probe block co-polymer lithography (SPBCL) to fabricate precisely positioned single metallic NPs with control over the particle size,^27–30^ we hypothesized the use of SPBCL to fabricate hG with enhanced hole distribution. SPBCL might offer an exquisite control over the size and position of the resulting nanoholes on graphene surfaces (Scheme 1).

Graphene hydrophobicity,^31^ thermal stability (≤600 °C),^32^ and electrical conductivity^33^ are the determinant properties that could permit the use of SPBCL to fabricate uniformly distributed AgNPs on its surface. Subsequent chemical etching of patterned AgNPs is expected to create patterned nanoholes on graphene with high uniformity.

## Experimental

### Materials

Dettol hand soap (DHS) (original) was bought from a nearby grocery store. Ethanol (EtOH) and hexane were purchased from Fisher Scientific, Ltd., UK. Graphene substrates (monolayer graphene on Si/SiO_2_ substrates (2.5 × 2.5 cm^2^)) were obtained from Graphena, Inc., Spain. Graphite in powder form was purchased from Sigma-Aldrich, USA. HNO_3_ was bought from Panreac Química SLU, Spain. Hexamethyldisilazane (HMDS) was obtained from Alfa Aesar, USA. Poly(ethylene oxide-*b*-2-vinylpyridine) (PEO-*b*-P2VP) (*M*_n_: PEO 2800 and P2VP 1500) was obtained from Polymer Source, Inc., Canada. Polyvinylpyrrolidone (PVP) (*M*_w_: 1 300 000) and silver nitrate (AgNO_3_) were purchased from Sigma-Aldrich, USA. Milli-Q® distilled deionized water (DDW) was used throughout the course of the investigation.

### Printing process

Si/SiO_2_ substrates (2 × 2 cm^2^) and polydimethyl siloxane (PDMS) pen arrays with a 100 μm tip-spacing were purchased from TERA-Print, LLC., USA. In a typical experiment, a Si/SiO_2_ substrate was vapor primed with an HMDS : hexane mixture (1 : 1 v/v) for 24 h under static vacuum to render the surface hydrophobic.^34^ Following a procedure developed by Noroozi *et al.*^35^ with slight modification, graphene was exfoliated out of graphite (1 mg mL^−1^) upon mixing with PVP (0.9 mg mL^−1^) and DHS (12.50 μL mL^−1^) in DDW (4 mL) with the aid of probe sonication in a continuous mode at 60% amplitude at various sonication times (1–4 h). To self-assemble graphene flakes into a uniform layer, graphene in PVP : DHS (1 mL) was diluted with EtOH (5 mL), sonicated for 15 min, and then injected in a timely manner into DDW (12 mL). The self-assembled layer was next carefully transferred to the HMDS-primed substrate and allowed to dry under ambient conditions. The ink was prepared *via* dissolving PEO-*b*-P2VP (5 mg mL^−1^) in DDW under continuous stirring till a transparent solution was obtained. Subsequently, AgNO_3_ (0.5 mM) was added and the pH was adjusted to 3 using HNO_3_. The ink was kept under stirring for 3 h in the dark prior to the electrospraying process. A PDMS pen array was plasma cleaned using a Zepto plasma cleaner (Diener Electronic-Germany, O_2_ plasma, 18 psi, 120 W, 220 s) directly before ink deposition *via* electrospraying using a Spraybase® instrument (Spraybase®-Ireland, emitter inner diameter: 0.35 mm, flow rate: 1 mL h^−1^, distance from the injector to the collector: 10 cm, voltage: 8.4 kV, and duration of electrospraying: 10 min). SPBCL experiments were carried out on a TERA-Fab M series instrument, TERA-Print, LLC., USA, operating at a temperature of 27 ± 2 °C and a relative humidity (RH) of 92 ± 2%. Patterns of 12 × 12 dots were created with a step size of either 0.5 μm or 1 μm and variable dwell times (s) and *z*-piezo extensions (μm) while keeping the printing force constant at 200 mN. Annealing was carried out using a tube furnace (Lindberg/Blue M™ 1200 °C Split-Hinge, Thermo Scientific™, USA) having multiple gas ports to attach cylinders of Ar and H_2_.

### Chemical etching process

Each printed substrate was dipped in an HNO_3_ aqueous solution (2.5 M), sonicated, and shaken for predetermined periods of time, after which the substrate was allowed to dry under ambient conditions.

### Characterization

The samples were analysed using the following characterization techniques: UV-Vis spectroscopy (PerkinElmer spectrometer-Lambda 950), thermogravimetric analysis (TGA) (NETZCH 209 F1 Thermo-gravimetric analyzer), Raman spectroscopy (Thermo DXR3 Raman Microscope), and scanning electron microscopy (SEM) (JEOL SEM-JSM-IT500HR-equipped with different detectors: an energy dispersive X-ray spectroscopy (EDS) detector, secondary electron detector (SED), and backscattering detector (BED)). The EDS detector is used to measure the spectrum of generated X-rays to determine the elements present in the sample (qualitative analysis) along with tracking the amount of each element from the intensity of the characteristic X-ray signals (quantitative analysis). The BED conveys information on the sample's composition wherein the number of backscattered electrons reaching the detector is proportional to the atom's *Z* (atomic) number. Higher *Z* atoms scatter more electrons back towards the detector than lower *Z* atoms and thus create higher signals. For example, the heavier Ag atoms (*Z* = 47) scatter more electrons than the lighter C (*Z* = 6) or O (*Z* = 8) atoms and thus appear brighter in the SEM images recorded using the BED. In contrast, the SED conveys topographic information of the sample surface as it detects secondary electrons originating from the surface or near-surface regions of the sample owing to the inelastic interactions between the primary electron beam and the sample.

### Image processing

Each SEM image was processed using the SEM software (SEM Operation), wherein the ruler tool was used to quantitatively measure the sizes of all AgNPs/formed holes. A line was drawn by tapping the start point of the target NP/hole and dragging the line to the end point of the target. Once done, the size will be displayed. This process was repeated for all NPs/holes in order to calculate the average size and standard deviation. The relative area of holes to that of graphene was calculated using ImageJ software.

## Results and discussion

SPBCL is increasingly becoming a preferred route to fabricate metal NPs owing to its vast capabilities to exquisitely control the size, shape, and position of NPs on solid substrates. SPBCL-defined polymeric nanoreactors govern the size of fabricated features *via* confining NP nucleation and growth, which are primarily affected by the hydrophobicity of the substrate, the printing force, *z*-piezo extension, and dwell time along with the conditions utilized during the subsequent thermal annealing step.^36^

**Fig. 1:**
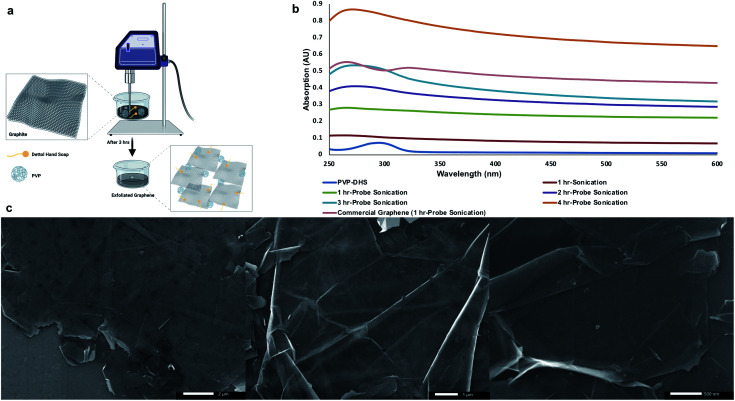
(a) Schematic of graphene exfoliation out of graphite using a DHS : PVP aqueous dispersion with the aid of probe sonication, (b) UV-Vis spectra of graphene dispersions obtained at different probe sonication times, and (c) SEM micrographs of the Marangoni self-assembly of graphene.

Si/SiO_2_ substrates were primed with HMDS *via* vapor coating of an HMDS : hexane mixture to ensure substrate hydrophobicity.^34^ To uniformly deposit graphene flakes on HMDS-primed substrates, graphene was first exfoliated out of graphite following a procedure developed by Noroozi *et al.*^35^ with slight modification. Briefly, graphite was suspended in a PVP : DHS aqueous dispersion with the aid of probe sonication as shown in Fig. 1a. Probe sonication was carried out in a continuous mode at 60% amplitude at various sonication times (1–4 h) to examine the output graphene flakes against a commercially available graphene suspended in the PVP : DHS aqueous dispersion and probe sonicated for 1 h. The UV-Vis spectrum of commercial graphene shown in Fig. 1b revealed the characteristic absorbance peak of graphene centered around 270 nm corresponding to the π/π* transitions of the aromatic C=C bonds along with a shoulder around 320 nm attributed to n/π* transitions of C=O bonds present in graphene oxide.^37^ A gradual increase in absorption around 270 nm was found with increasing probe sonication time, 3 h of which exhibited a comparable absorbance to that of commercial graphene with a slight red-shifting indicating the preservation of the electronic conjugation within the graphene basal plane.^38^ Thereby, 3 h-probe sonication was applied hereafter to obtain exfoliated graphene flakes in the PVP : DHS aqueous dispersion. Next, a uniform layer of graphene flakes was deposited on the HMDS-primed substrate *via* Marangoni self-assembly described by Ye *et al.*^39^ Briefly, graphene in PVP : DHS was diluted with EtOH and sonicated for 15 min to ensure the uniformity of graphene flakes, which allowed their injection in a timely manner into DDW. Besides graphene hydrophobicity, the difference in surface tension between EtOH and DDW permitted graphene flakes to float, collide, and self-assemble to ultimately form a continuous layer of graphene in a uniform fashion. This layer was carefully transferred to the HMDS-primed substrate and allowed to dry under ambient conditions (Fig. 1c) in order to be subsequently used for the SPBCL experiment.

In parallel to substrate preparation, the ink was prepared *via* dissolving PEO-*b*-P2VP in DDW at a concentration of 5 mg mL^−1^ followed by the addition of AgNO_3_ (0.5 mM). The pH was adjusted to 3 using HNO_3_, and the ink was kept under stirring in the dark for 3 h to ensure the complete coordination between the pyridine group and Ag ions. PEO-*b*-P2VP is expected to form micelles in DDW with P2VP core and PEO shell. The hydrophobic core coordinates with metal precursors while the hydrophilic shell ensures ink fluidity during printing in a high humidity environment. The ink was next electrosprayed on the polymer pen array and allowed to dry prior to mounting on the instrument. The sprayed polymer pen array was incubated for 2 hours in a high humidity environment (92 ± 2%) at 27 ± 2 °C prior to the printing process. This step is necessary to enable ink hydration, spreading, and diffusion, thereby facilitating its transport to the substrate. A 12 × 12 dot matrix design was used to print the ink using 200 mN of force, 1 s of dwell time, and a step size of 500 nm. Printing and patterning parameters are summarized in Table 1.

**Table 1:** Instrumental parameters used for printing the ink *via* SPBCL on Marangoni self-assembled graphene flakes on the HMDS-primed substrate

Printing parameters		Patterning parameter		
Force	200 mN		*X*	*Y*
Voltage	10 V	Start point	5 μm	5 μm
Current threshold	0.2 mA	Step size	500 nm	500 nm
Dwell time	1 s	No. of points	12	12
Relative *z*-piezo extension	0 μm	Spacing between pens	100 μm	100 μm

**Fig. 2:**
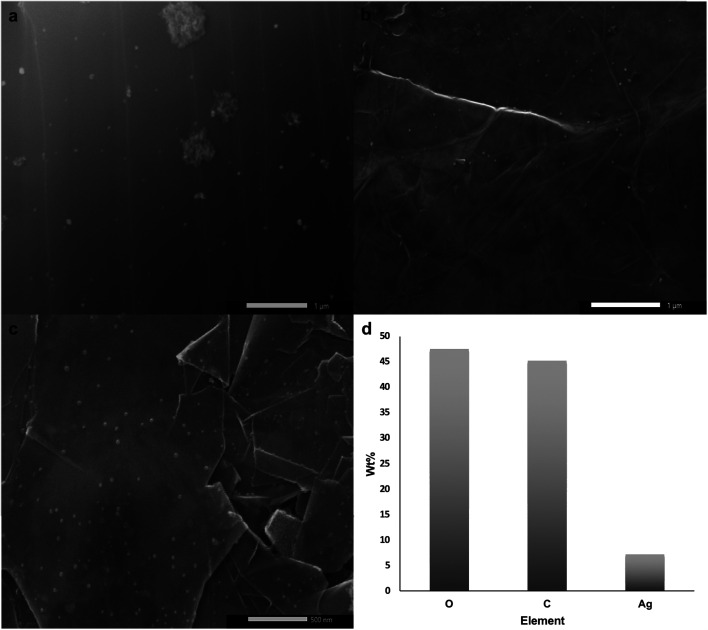
(a–c) SEM micrographs of the uniformly distributed AgNPs on graphene flakes, and (d) EDS analysis of Ag–graphene flakes.

**Fig. 3:**
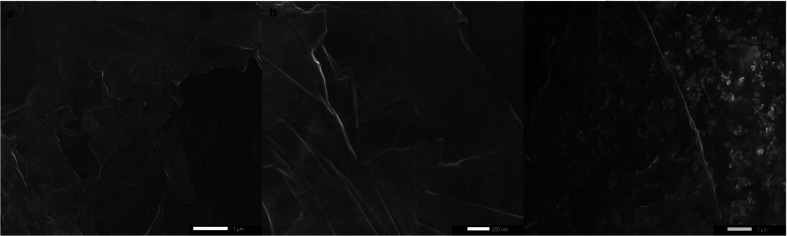
SEM micrographs of etched AgNP-decorated graphene after (a and b) sonication for 6 h, and (c) mechanical agitation for a week.

The printed substrate was thermally annealed at 150 °C for 16 h under Ar atmosphere and then at 500 °C for another 16 h under H_2_. The first annealing stage induced AgNO_3_ particle nucleation and coarsening as the decomposition of AgNO_3_ wouldn't start unless the temperature reaches ∼285 °C. The second annealing stage initiated reduction and particle growth producing AgNPs^29^ along with decomposition of PEO-*b*-P2VP, PVP, and DHS since their decomposition temperatures are 409 °C,^40^ 436 °C, and 289 °C (Fig. S1†), respectively. SEM micrographs shown in Fig. 2a–c reveal the uniform size distribution of AgNPs with an average of 38 ± 13 nm on graphene flakes. EDS analysis confirmed the presence of elemental Ag as shown in Fig. 2d. Subsequent chemical etching of AgNPs using HNO_3_ (2.5 M) under sonication for 6 h resulted in no obvious hole formation probably because the holes were too small to be seen and/or the contrast was too weak as shown in Fig. 3a and b. Thus, mechanical agitation was carried out for a week while monitoring the hole formation on a daily basis. On day 7, hG with uniformly distributed holes with an average size of 152 ± 45 nm was produced (Fig. 3c).

AgNPs acted as a catalyst to oxidize the surrounding carbon atoms resulting in CO/CO_2_ liberation and hole generation. This is generally accompanied by ‘track-marks’ formation owing to the etching-induced motion of AgNPs.^12,13^ Apparently, the holes' size is 4× larger than the size of AgNPs, which can be attributed to hole merging and/or excessive oxidative etching owing to the prolonged etching time.

**Fig. 4:**
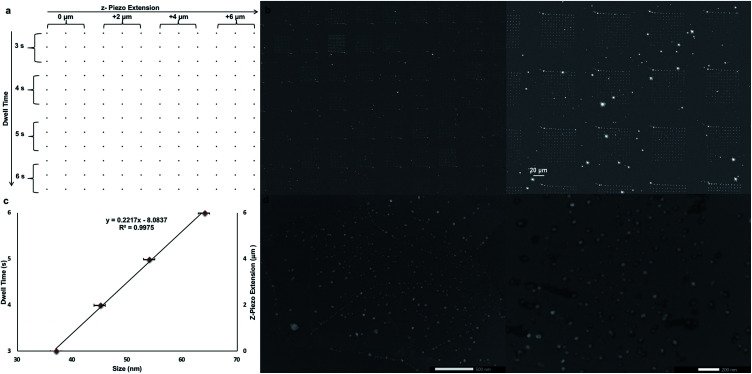
(a) Pattern of the dot matrix used for printing the ink dispersion on a CVD graphene substrate at 92 ± 2% RH and 27 ± 2 °C, (b) light microscope images of the CVD-graphene substrate right after ink printing, (c) plot representing the sizes of fabricated AgNPs on the CVD-graphene substrate upon varying the relative *z*-piezo extension and dwell time, and (d) fabricated AgNPs on the CVD-graphene substrate.

After proven successful, the SPBCL approach was re-utilized to fabricate AgNPs of tunable sizes on a CVD-graphene substrate in order to lessen the number of steps and simplify the whole process. The same methodology for ink preparation, electrospraying, and humidification was followed. Yet, a 12 × 12 dot matrix design was used to print the ink dispersion *via* applying 200 mN of force, varied dwell times (3–6 s), varied relative *z*-piezo extensions (0–6 μm), and a step size of 1 μm in both *X* & *Y* directions as shown in Fig. 4a and summarized in Table 2. These variations in *z*-piezo extensions and dwell times would influence the size of the resulting AgNPs; the (0 μm, 3 s) block would result in fabricating the smallest NPs whereas the (6 μm, 6 s) block would account for the largest NPs.

**Table 2:** Instrumental parameters used for printing the ink *via* SPBCL on a CVD-graphene substrate

Printing parameters		Patterning parameter		
Force	200 mN		*X*	*Y*
Voltage	10 V	Start point	5 μm	5 μm
Current threshold	0.2 mA	Step size	1 μm	1 μm
Dwell time	3–6 s	No. of points	12	12
Relative *z*-piezo extension	0–6 μm	Spacing between pens	100 μm	100 μm

These instrumental parameters could have a pronounced effect on the amount of delivered ink to the substrate; increasing the relative *z*-piezo extension could increase the tip-substrate contact area and thereby increase the amount of delivered ink. Parallel to this, prolonged dwell times deliver larger amounts of ink to the substrate. Thereby, a tunable gradient of feature sizes was fabricated *via* varying the relative *z*-piezo extension and dwell time as evidenced in Fig. 4b. The printed CVD-graphene substrate was next thermally annealed under Ar atmosphere at 200 °C for 10 h and then at 500 °C for another 6 h. Four main populations of NPs ranging between 37 ± 3 nm and 64 ± 3 nm were observed owing to the applied variations in both the *z*-piezo extension and dwell time (Fig. 4c). Apparently, the fabricated AgNPs were of uniform size distribution on the CVD-graphene substrate showing no signs of aggregation (Fig. 4d). Indeed, the SPBCL approach adopted here evokes exquisite control over the size of the fabricated NPs on the CVD-graphene substrate *via* controlling the printing force, *z*-piezo extension, and dwell time.

**Fig. 5:**
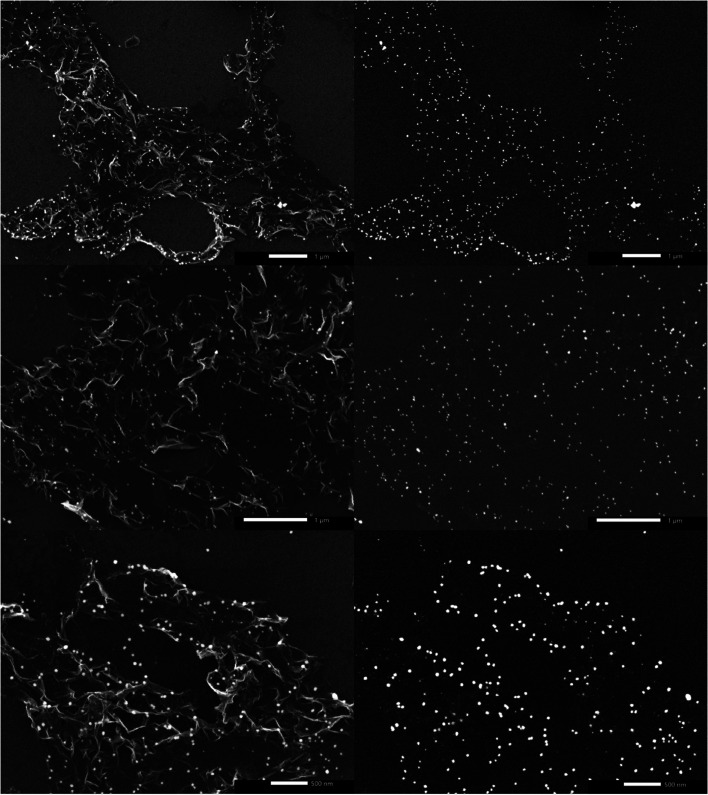
SEM micrographs of the broken CVD-graphene sheet into smaller structures containing AgNPs owing to sonomechanical treatment in the presence of HNO_3_. Images on the left were taken using the secondary electron detector (SED) whereas those on the right are the representative images taken using the backscattering detector (BED).

Despite their uniform size distribution, AgNPs lost their patterned spatial features. This can be attributed to the partial loss of graphene hydrophobicity in the thermal annealing step, leading to the formation of multiple NPs per nanoreactor. Boosting graphene hydrophobicity *via* chemical means prior to the printing process might tremendously help in overcoming this encountered challenge. Another obstacle is the presence of defects in the CVD-graphene substrate including grafolds, in which AgNPs were trapped resembling the beads in a chain. Subsequent etching of AgNPs using HNO_3_ (2.5 M) with the aid of sonication for 4 h and shaking for another 14 h helped with the guided breakage of the graphene sheet into smaller structures owing to the presence of catalytic AgNP chains specifically in the grafolds (Fig. 5).

**Fig. 6:**
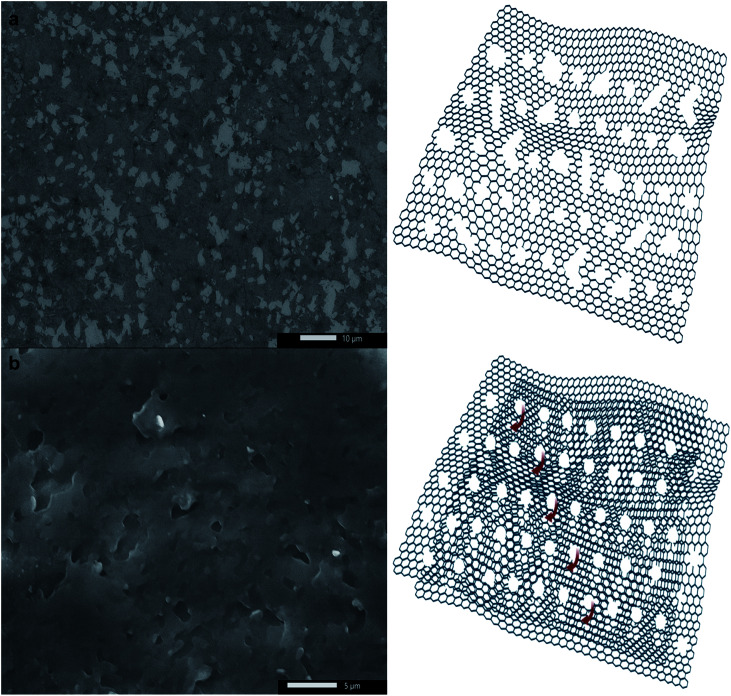
SEM micrographs (left) and schematics (right) of (a) an hG sheet, and (b) two-dimensional laminar porous graphene.

Prolonged etching (8 h of sonication and 28 h of shaking) produced either hG sheets (Fig. 6a) or two-dimensional laminar porous graphene (Fig. 6b) owing to the removal of AgNPs producing holes and subsequently stacked hG sheets. Similar to hG flakes, holes could be observed in both hG sheets and the two-dimensional laminar porous graphene exhibiting four main populations ranging between 207 ± 43 nm and 411 ± 63 nm. The relative area of holes to that of the graphene sheet was calculated to be 85 ± 8%.

**Fig. 7:**
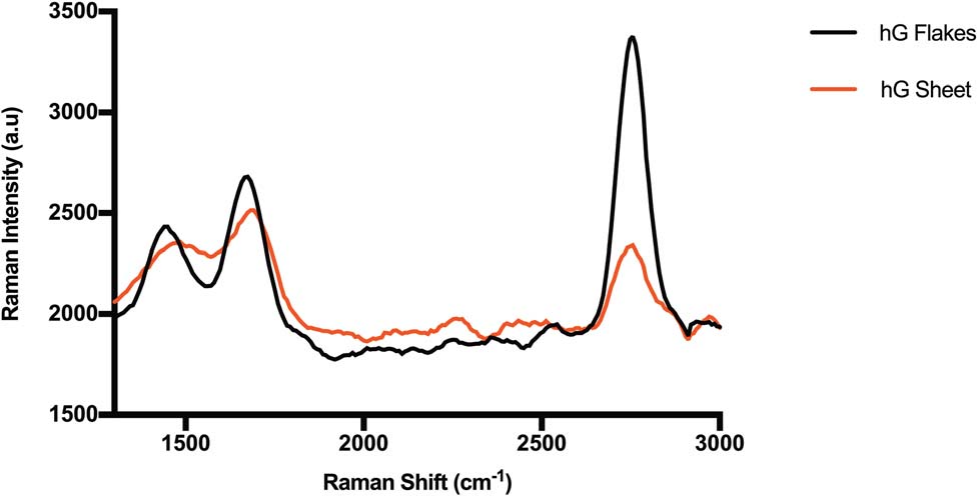
Raman spectra of hG flakes and an hG sheet.

Despite their holey structures, both hG flakes and sheets considerably retained their two-dimensional graphitic crystallinity as evidenced in Fig. 7. Raman spectra showed a D band at ∼1420 cm^−1^, a G band at ∼1630 cm^−1^, and a 2D band at ∼2763 cm^−1^. The obtained *I*_D_/*I*_G_ ratios for hG flakes (0.89) and hG sheets (0.96) were lower than those commonly reported for GO (*I*_D_/*I*_G_ > 1). This plausibly stemmed from removing more defective C atoms during the etching process to produce hG while preserving the remaining C atoms in ordered or crystalline structures despite the increase in the hole diameter.^41,42^

The graphene surface could be primed with *N*-methyl-2-pyrrolidone (NMP) followed by HMDS^43^ to protect its conductivity during the printing step as well as its hydrophobicity during thermal annealing. This would further enhance the size and distribution of AgNPs on the graphene surface, if needed. The downstream etching step could be optimized to improve the size of holes *via* adjusting the sonication/shaking time along with the HNO_3_ concentration or employing oxygen plasma as an alternative etchant.

## Conclusion

Graphene physiochemical properties including hydrophobicity, thermal stability, and electrical conductivity permitted the use of SPBCL to fabricate uniformly distributed AgNPs on its surface with minimal to no aggregation. Regardless of all encountered challenges, the developed SPBCL approach elicited a precise control over the size and position of the fabricated AgNPs on the graphene surface and hence producing hG of uniform hole size distribution after HNO_3_-induced etching. Indeed, SPBCL could be used to fabricate custom-made metal NP–graphene composites and/or their etched counterparts with spatial and dimensional control, offering a powerful route to nanofabrication and nanomanipulation bypassing the implementation of conventional methods. The produced hG might exhibit promising properties featuring high conductivity and hence it can be used in electronics, energy storage devices, and electro-catalytic reactions. Also, it can be potentially applied in biomedical applications either in the detection or the treatment of diseases.

## Author contributions

S. A. A., E. H. A., and A. H. A. designed the research; S. A. A., R. S. F., and S. A. performed the research; S. A. A. and A. H. A. analyzed the data; and S. A. A., E. H. A., A. M. A., and A. H. A. wrote and finalized the manuscript.

## Conflicts of interest

There are no competing interests to declare.

## Supplementary Material

NA-004-D1NA00769F-s001

NA-004-D1NA00769F-s002

NA-004-D1NA00769F-s003
