# Supplementary material for: Custom-made holey graphene via scanning probe block co-polymer lithography
Source: Nanoscale Adv. 2022 Jan 31;4(5):1336–44. doi: 10.1039/d1na00769f (PMC9418674; doi:10.1039/d1na00769f)
Supplement: NA-004-D1NA00769F-s001 [file NA-004-D1NA00769F-s001.pdf]

Electronic Supplementary Material (ESI) for Nanoscale Advances.
This journal is © The Royal Society of Chemistry 2022

Supplementary Information

Custom-made Holey Graphene via Scanning Probe Block Co-polymer Lithography

Samar A. Alsudir[a], Roa S. Fardous[a], Shahla Alsoughayer [b], Abdulaziz M. Almalik[a,b], Edreese H. Alsharaeh[c], Ali H. Alhasan[*c,d]

[a]National Center for Pharmaceutical Technology, Life science and Environmental Research Institute, King Abdulaziz City for Science and Technology (KACST), P.O. Box 6086, Riyadh 11461, Saudi Arabia

[b]KACST-BWH/Harvard Centre of Excellence for Biomedicine, Joint Centers of Excellence Program, King Abdulaziz City for Science and Technology (KACST), P.O. Box 6086, Riyadh 11461, Saudi Arabia

[c]College of Science and General Studies, Alfaisal University, P.O. Box 50927, Riyadh 11533, Saudi Arabia

[d]National Center for Biotechnology, Life science and Environmental Research Institute, King Abdulaziz City for Science and Technology (KACST), P.O. Box 6086, Riyadh 11461, Saudi Arabia

*Corresponding author: Ali H. Alhasan

Email: aalhasan@kacst.edu.sa

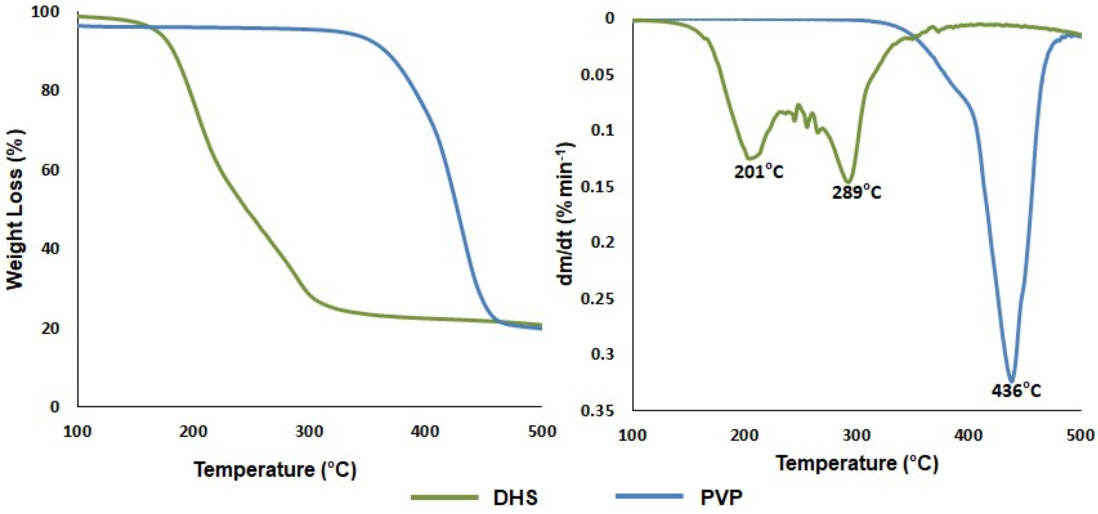

Figure S1: Graphs of thermogravimetric analysis (TGA) determining the decomposition temperatures of PVP and DHS.
