# Supplementary figures and images for: Custom-made holey graphene via scanning probe block co-polymer lithography
Source: Nanoscale Adv. 2022 Jan 31;4(5):1336–44. doi: 10.1039/d1na00769f (PMC9418674; doi:10.1039/d1na00769f)

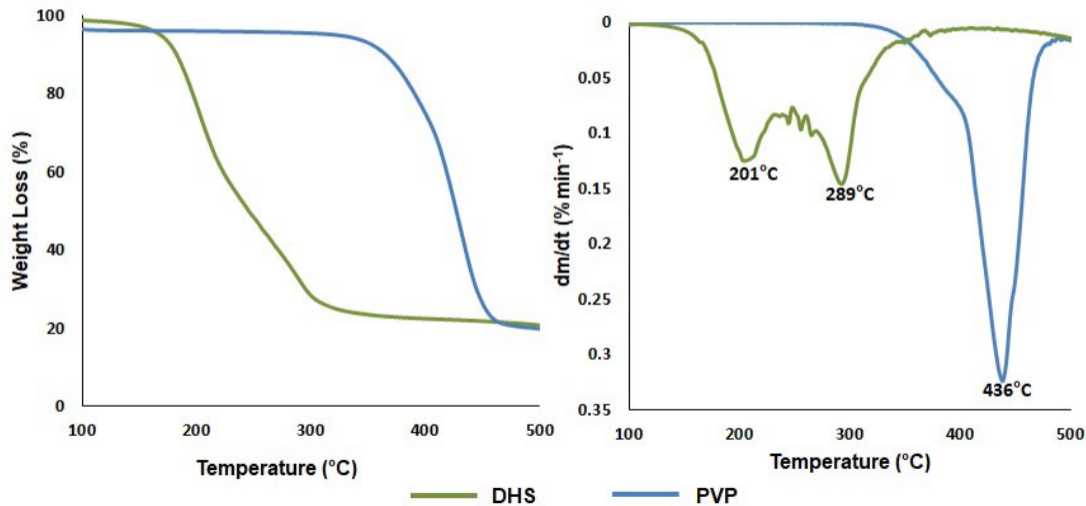

Supplement: NA-004-D1NA00769F-s003 [file NA-004-D1NA00769F-s003.pdf]
